# Psychological Resilience Buffers Depression and Post-Traumatic Stress Disorder Due to Childhood Trauma in Thai Seniors

**DOI:** 10.3390/medicina61081355

**Published:** 2025-07-26

**Authors:** Moe Moe Yu, Kanokporn Pinyopornpanish, Nahathai Wongpakaran, Ronald O’Donnell, Tinakon Wongpakaran

**Affiliations:** 1 Mental Health Program, Multidisciplinary and Interdisciplinary School, Chiang Mai University, Chiang Mai 50200, Thailand; moemoeyu_m@cmu.ac.th (M.M.Y.); nahathai.wongpakaran@cmu.ac.th (N.W.); 2 Department of Family Medicine, Faculty of Medicine, Chiang Mai University, Chiang Mai 50200, Thailand; kanokporn.pinyopo@cmu.ac.th; 3 Department of Psychiatry, Faculty of Medicine, Chiang Mai University, Chiang Mai 50200, Thailand; 4 Behavioral Health, College of Health Solutions, Arizona State University, Tempe, AZ 85004, USA; ronald.odonnell@asu.edu

**Keywords:** childhood trauma, resilience, depressive symptoms, PTSD, older adults

## Abstract

*Background and Objectives*: Thailand’s demographic shift toward an aging population increases vulnerability among older adults to the long-term mental health effects of childhood trauma. While childhood adversity is linked to heightened risks of late-life depression and PTSD, the moderating role of resilience remains underexplored in Thai older adults. This study investigated whether resilience moderates the association between childhood trauma and depressive or PTSD symptoms in this population. *Materials and Methods*: A cross-sectional survey was conducted with 201 older patients (mean age 68.6 years) from the Family Medicine and Geriatric Psychiatry Clinics at Maharaj Nakorn Chiang Mai Hospital. Participants completed validated measures on trauma history, resilience, depression, and PTSD symptoms. A moderation analysis was performed, adjusting for covariates including education, family support, and psychiatric history. *Results*: The findings revealed that resilience significantly buffered the impact of childhood trauma on depression but did not mitigate PTSD symptoms. *Conclusions*: These results underscore the protective role of resilience against depression following childhood trauma in older adults, yet also point to its limitations in alleviating trauma-specific responses such as PTSD. This study highlights the need for trauma-informed geriatric mental health strategies and calls for further research on resilience, focusing on cultural context and adaptive mechanisms, including emotion regulation and social connectedness, in older populations.

## 1. Introduction

Thailand, like many ASEAN countries, is undergoing a demographic shift toward an aging population, now ranking second after Singapore, with older adults comprising 19%—a figure expected to exceed 30% within 15 years [1]. Aging often brings declines in physical and psychological resilience, and the erosion of meaningful social roles [2,3]. Against this backdrop of increasing vulnerability in later life, it is essential to recognize that experiences from earlier in the lifespan—particularly those involving adversity—can have enduring effects that persist into old age. 

Among the adversities across lifespan, childhood trauma is increasingly recognized as a critical determinant influencing long-term mental health consequences. Over half of older adults report having experienced at least one adverse childhood experience (ACE), including abuse, neglect, and family dysfunctions [4,5,6,7]. Neurobiological studies suggest that these long-term consequences may be mediated by alterations in the hypothalamic–pituitary–adrenal (HPA) axis and structural changes in regions such as the hippocampus and amygdala [8,9,10,11,12]. Furthermore, individuals with early trauma histories often perceived greater stress when encountering later-life stressors and tended to respond poorly to treatment [11,13]. The unresolved traumatic experiences that older adults face increase the risk of mental health conditions such as depression and post-traumatic stress disorder [4,7]. 

Trauma is not confined to early life. Lifetime trauma—as defined in the DSM-5 as exposure to or witnessing severe physical or psychological events—also influences mental health outcomes in older age [14,15,16]. In Thailand, events such as natural disasters and the COVID-19 pandemic have had substantial psychological impacts, with studies reporting probable PTSD prevalence ranging from 12.6% to 44.5% among flood victims [17,18,19].

Childhood trauma has been shown to predict the severity and persistence of late-life depressive symptoms [20], with depression affecting 31.7% to 35.1% of older adults globally [21,22], and 23.7% in Thailand as of 2019 [23], aligning with figures from other cultural contexts [24]. Depression in late life is associated with increased multimorbidity, suicidal ideation, and mortality yet remains underdiagnosed and undertreated [25]. Similarly, PTSD, is linked to both psychological and physical comorbidities, including cardiovascular and gastrointestinal issues [3,26]. Among older Thai adults in long-term care, 5.5% met the criteria for PTSD during the pandemic, with key risk factors including strained family relationships and financial hardship [27]. PTSD risk is further shaped by individual and contextual factors such as gender, social support, and cumulative trauma exposure [7,28,29,30,31]. 

Growing evidence connects ACEs, including abusive parent–child relationships, to persistent psychiatric risks across the lifespan, including depression, anxiety, PTSD, and even early mortality [4,6,7,32,33,34,35,36,37]. Beyond psychological effects, childhood adversity is associated with greater physical frailty, chronic illness, cognitive decline, and sleep disturbances in older age [29,38,39,40,41,42,43]. Despite the strength of these associations, the trauma research on older adults in Thailand remains scarce, particularly regarding its links to depressive and PTSD symptoms. Given this gap, attention has increasingly turned to factors that may buffer or moderate the effects of early adversity, chief among them of which is resilience.

Resilience, conceptualized as the capacity to maintain or regain psychological well-being despite adversity, has emerged as a key factor in trauma adaptation. It is shaped by personality traits, social and economic resources, and stress exposure [44,45]. Stress inoculation theory posits that early, manageable, or non-traumatic stress may enhance adult resilience [46], though childhood adversity, especially emotional abuse and neglect, appeared to have negative associations with later resilience [47]. Moreover, adaptive functioning requires interconnected systems within the social environment [48,49]. The resilience portfolio model expands on this by emphasizing strengths in regulation, interpersonal relationships, and meaning-making as critical for trauma recovery [50]. However, resilience does not equate to immunity from psychopathology [45]. For instance, high resilience may coexist with elevated anxiety and hyperarousal stress, as seen during the pandemic in Iran [51]. Nonetheless, meta-analyses confirm that resilience negatively correlates with depression and PTSD [52,53] and moderates the impact of perceived stress in later life [54].

**Figure 1:**
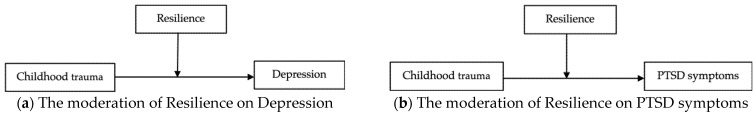
The proposed moderation model.

Despite increasing global interest in these dynamics, few studies have investigated the interplay of childhood trauma, resilience, and psychopathology among older Thai populations. To address this gap, the present study examines whether resilience moderates the relationship between childhood trauma and depressive or PTSD symptoms in older adults attending tertiary primary care settings. We hypothesize that greater resilience may buffer the adverse mental health effects of childhood trauma. Figure 1 shows the moderation model of resilience between childhood trauma and (a) depression (b) PTSD symptoms.

## 2. Materials and Methods

### 2.1. Study Design

To examine the prevalence and relationships among study variables, the researchers designed a cross-sectional study that collected data from Thai older patients.

### 2.2. Participants and Setting

We enrolled a sample of older patients who visited the geriatric Family Medicine and Psychiatry clinics of Maharaj Nakhon Chiang Mai Hospital between October and December 2024. Due to the exploratory nature of the research, convenience sampling was employed across multiple primary care settings, ensuring demographic representativeness. We obtained efficient data from the target population within a limited study period in clinical settings [55]. Ethics approval was obtained from the Faculty of Medicine, Chiang Mai University, Thailand (study code, PSY-2567-0388).

Sample size calculation for moderation analysis was performed with a medium effect size, a power (β) set to 0.8, and a significance level (α) of 0.05. The minimum sample size required for analysis was 118. However, a total sample of 201 participants was collected and analyzed in this study.

The inclusion criteria were age 60 years and above, the ability to communicate in Thai both verbally and in writing, and the ability to be reached in person or by phone. Patients who were critically ill; had severe psychiatric conditions sufficient to interfere with their participation in the study such as disorientation, dyspnea, severe substance addictions, manic episodes, and psychotic disorders; were currently receiving treatment for psychosis; had moderate to severe physical disabilities (e.g., loss of eyesight or hearing); had major neuro-cognitive disorders; or could not complete the questionnaires were excluded.

After obtaining ethical approval, the study announcement for recruiting volunteers was posted at the clinics using posters. At the mentioned OPDs, a Thai-speaking research assistant and the researcher waited for the convenience and availability of potential patients during their visits. When staff from the clinic identified patients who were eligible and met the inclusion criteria, the Thai research assistant approached them to explain the study and invite them to participate. After agreeing to participate and obtaining informed consent by signing the form, participants began their involvement in the study by completing the questionnaire. Participants were compensated 100 baht (approximately USD 2.97) upon completion of the survey. The OPD staff offered a private space within clinics, i.e., some teaching rooms or small clinic rooms, for participants joining the study.

### 2.3. Measurements

The questionnaire comprised several measures, beginning with a self-developed questionnaire to collect sociodemographic and health condition data. Then, it continued to questions related to traumatic events in childhood and lifetime, the Resilience Inventory, the Thai Geriatric Depression Scale, and the PTSD symptoms checklist.

#### 2.3.1. Self-Developed Sociodemographic and Health Condition Questionnaire

Sociodemographic information about age, sex, religion, education, marital status, number of household members, living status, perceived family relationship, monthly income, and health scheme (government official/30-baht/social security/health insurance, and self-paid) were collected together with health condition questions such as diagnosed chronic disease and psychiatric condition, family history of psychiatric condition, alcohol consumption, substance use (smoking/tobacco or cannabis) in the past month, and recent history of fall.

#### 2.3.2. Instruments

##### Modified Traumatic Experience Scale (TES) for Childhood Trauma

TES is a self-report questionnaire to measure childhood traumatic experiences. A modified scale was added to the original scale for sexual and household dysfunction aspects, which included physical and emotional aspects. The response format was dichotomous, simply to choose ‘Yes’ or ‘No’. The score is the summation of experience counts. Reverse questions were included and counted reversely. The original TES aligns well with the Rasch measurement model, exhibiting sufficient unidimensionality (first contrast = 1.80) and a disattenuated correlation between person measures of 0.8282. The Infit/Outfit Mean Square (MNSQ) values range from 0.71 to 1.52. The Person Separation Index is 3.3, with a Person Reliability of 0.81 and Cronbach’s alpha of 0.92. Additionally, the Item Separation Index is 3.04, and the Item Reliability is 0.90 [56].

##### Lifetime Traumatic Experience

The questions for lifetime traumatic experience are referred to as Criteria-A for the diagnosis of Post-Traumatic Stress Disorder in DSM-5 [14]. The list of traumatic events was provided in dichotomous format, with YES or NO answers, together with the time of the event, whether experienced within or more than 1 month ago. Participants were also asked if any other traumatic event not listed was included, and an open-ended question was provided for them to write about it. The list was added with follow-up questions, i.e., no. of exposures and age at first exposure [57]. The score will be the sum of events experienced.

##### 9-Item Resilience Inventory (RI-9)

RI-9 is a 9-item self-report questionnaire measuring a person’s resilience in terms of self-efficacy, self-reliance, self-awareness, and adaptability. For example, “I can withstand the pressure” and “I believe that in times of crisis, there is always an opportunity”. Each item has a 5-point Likert scale, ranging from 1 to 5, with 1 representing the lowest level of agreement and 5 representing the highest level. The interpretation of scoring is simple by summation: the higher the score, the higher the level of resilience. The reliability analysis of RI-9 was 0.86. The RI-9 scales correlated with ten scales of the Inner Strength-Based Inventory (ISBI), confirming its convergent validity. Cronbach’s alpha was 0.90–0.97, with a sufficient sample [56,58].

##### Thai Geriatric Depression Scale-6 (TGDS-6)

TGDS-6 is used in screening depression in community dwellings and outpatient settings. TGDS-6 was derived from TGDS-15 in a short version for rapid screening purposes. TGDS-6 provided AUCs of >0.8, indicating good accuracy performance. There are two reverse questions included. The scale gives a sensitivity of 73.29% and a specificity of 81.24%. The tool is simple to use, with ‘YES’ and ‘NO’ response options for 6 questions. The cut-off point for depression is 2 [59].

##### PTSD Check List for Civilian (PCL-C)

The Thai version was translated from the original English version [60]. Participants were asked if they had experienced the symptoms mentioned for at least one month. PCL is used to assess symptoms of PTSD in Criteria B (re-experiencing), C (avoidance and numbing), and D (Dysphoric and anxious arousal) of DSM-IV-TR [14]. A person can report on a 5-point Likert scale, where 1 = ‘Not at all disturbing’ to 5 = ‘Extremely disturbing’. The score can be from 17 to 85. In older adults, previous studies [61,62] endorsed a score of 37 at optimum sensitivity and specificity to diagnose PTSD. Cronbach’s alpha was mid to high 0.90 s in a wide range of populations [63]. The cut-off of 42 points was used for the prevalence of PTSD among older residents from long-term care in Thailand [27].

### 2.4. Statistical Analysis

For descriptive statistics, we began calculating the frequencies and percentages of categorical variables, such as age, sex, and religion, as well as the means and standard deviations for continuous variables, including childhood traumatic experience scores, RI-9, and TGDS-6. This analysis provided a better understanding of the sample and the prevalence of probable depression. Because the total scores of measuring variables and sociodemographic data were not normally distributed, non-parametric analyses were employed. Mann–Whitney U tests were performed for dichotomous variables to compare two groups (e.g., sex, religion, having psychiatric comorbidities, having a history of falls, etc.). Kruskal–Wallis tests were performed for variables with three or more levels (e.g., age, marital status, monthly income, etc.).

Correlation analyses were used to detect the correlations between variables. Spearman’s Rho correlations were performed for continuous and ordinal variables, and point biserial correlations were performed for dichotomous variables. The moderation was tested with the independent variable and the moderator, both defined as mean-centered, and the covariates were dichotomized. We applied a bootstrapping method (5000 samples) in the moderation analysis to enhance the accuracy of the results and calculate more reliable confidence intervals for correlations, given that our data consisted of only 201 samples and was non-normally distributed.

Statistical significance of all analyses was conducted at a significance threshold of *p* < 0.05. IBM SPSS, Version 26 (IBM Corp., Armonk, NY, USA), and PROCESS version 4.2 were used for analysis.

## 3. Results

### 3.1. Descriptive Analysis

Table 1 presents the descriptive statistics summarizing the distribution of study variables. Of the 201 participants, 70.1% were women, with a mean age of 68.56 years (SD = 5.009). Fewer than half had attained at least a secondary-level education. Approximately 54% resided in households comprising three or fewer individuals.

Regarding family relationships, 20.9% of participants characterized theirs as “average,” while the majority (79.1%) reported having a “good” family relationship. A family history of psychiatric conditions was reported by 8% of respondents. The prevalence of smoking, tobacco, or cannabis use was 4.5%, and 6% had a history of two or more psychiatric diagnoses. Additionally, 3% reported experiencing a fall in the past month.

The mean scores of resilience were moderately high. Childhood trauma was reported by 40.3% of participants. Specific types of childhood trauma—including physical, emotional, and sexual abuse—were each reported by at least 1% of the sample, with the exceptions of two categories, “serious physical abuse requiring hospitalization” and “sexual abuse by a stranger”, which were reported by fewer than 1%. Group differences across various covariates are further detailed in Table 1.

### 3.2. Difference Within Covariates

Group comparisons were performed using the *t*-test and ANOVA, and the results are summarized in Table 2.

Significantly higher resilience was observed in males and those with higher education or income; participants with good family relationships had lower depression and PTSD scores. The highest income group (>10,000 THB) showed the highest resilience and lowest depression scores. A family history of psychiatric conditions was associated with elevated PTSD symptoms. Recent tobacco use, falls, and psychiatric comorbidities were linked to higher depression scores, while a history of psychiatric comorbidities was also related to higher PTSD scores. Individuals exposed to at least one childhood trauma reported higher depression scores, and those with at least one lifetime trauma had higher depression and PTSD scores.

No significant differences were found across age, religion, marital status, alcohol use, or history of chronic illness. See Table 2 for details.

### 3.3. Correlation Analysis

Correlations among variables were analyzed by utilizing Spearman’s rank, Pearson’s, and point-biserial correlations. Refer to Table 3, Table 4 and Table 5. Sociodemographic data, medical and psychiatric history, and measurement scores were analyzed to find meaningful correlations.

Older age was associated with higher reports of lifetime trauma. Males generally had higher education, income, and resilience and better family relationships. Higher education and income were each linked to greater resilience, with higher income also associated with better family relationships and lower depression scores. Living alone and average family relationships correlated with more childhood trauma and lower income.

Alcohol use was more common among males with higher incomes. Smoking or tobacco use was correlated with cannabis use and higher depression, and cannabis use was also associated with depression. Childhood trauma was linked to living alone, average family relationships, depression, and PTSD symptoms, while lifetime trauma was related to older age, depression, and PTSD symptoms.

Higher resilience was associated with male sex and higher educational attainment. Depression was associated with lower income, average family relationships, substance use, greater trauma exposure, PTSD symptoms, and lower resilience. PTSD symptoms were associated with average family relationships, trauma exposure, family psychiatric history, recent falls, psychiatric comorbidities, and higher depression scores.

Religion showed no significant associations. See Table 3 for details.

Higher depression scores were linked to lower income, average family relationships, smoking/tobacco or cannabis use, greater childhood and lifetime trauma, and PTSD symptoms. PTSD scores were associated with average family relationship, family psychiatric history, history of recent falls, psychiatric comorbidities, both trauma types, and higher depression scores.

The family history of psychiatric conditions was significantly associated with falls, physical and psychiatric comorbidities, lifetime trauma, depression, and PTSD scores. Physical comorbidities were also linked to family psychiatric history. Psychiatric comorbidities were associated with the family psychiatric history, childhood trauma, depression, and PTSD scores.

The correlation analysis indicated meaningful associations between the study variables. Childhood and lifetime trauma were both positively linked to depression and PTSD symptoms. Resilience showed an inverse relationship with childhood trauma and depression, while demonstrating a subtle positive association with lifetime trauma and PTSD symptoms. A strong positive relationship was observed between depression and PTSD symptoms, highlighting considerable overlap in symptom presentation. These results support the study’s hypothesized patterns and lay the foundation for subsequent regression analyses.

### 3.4. Moderation Analysis

First, we tested the raw regression analysis by using linear regression on SPSS version 26. It resulted in childhood trauma being positively associated with depression. Second, we deployed childhood trauma (X) and resilience (W) at mean-centered products and tested the interaction term (X × W) on depression outcome without any inclusion of covariates. The interaction effect was significant at a 95% confidence level. Finally, when covariates continued to be included in the model, the interaction effect was found to be significant. Age, sex, education, marital status, monthly income, family relationship, family history of psychiatric conditions, having a history of physical comorbidity, alcohol consumption, smoking/tobacco use, history of fall in the past month, having recent/lifetime trauma, and having a history of psychiatric comorbidities were accounted for. Without the inclusion of the moderation effect, the R^2^ value for depression accounted for by childhood trauma was 10%; after the inclusion of the moderation effect (CT × RI), the R^2^ value became 14%. After the inclusion of new predictors, the model’s explained variance on the depression outcome increased by an additional 24%. See Table 6.

The addition of the interaction term in Model 2 explained an additional 2% of the variance in depression (∆*R*^2^ = 0.02, *f*^2^ = 0.023), indicating a small moderation effect. After including covariates in Model 3, the whole model explained that variance increased substantially by 24% (∆*R*^2^ = 0.24, *f*^2^ = 0.375), reflecting a significant overall effect. See Table 7. The formula used in the calculation of Cohen’s *f*-squared (Cohen’s *f*^2^) is shown here.

$$f^{2}=R^{2}full-R^{2}reduced/1-R^{2}full\;\;$$

**Figure 2:**
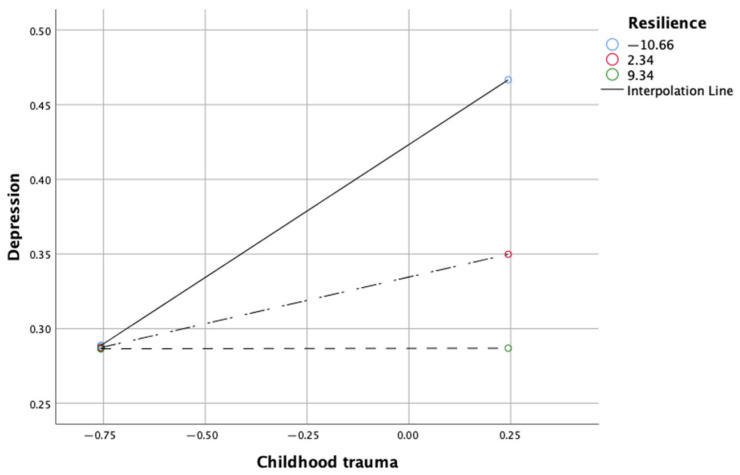
Simple slope analysis in testing the moderating effect of resiliency on the relationship between childhood trauma and depression.

The slope pattern for the moderation effect of resilience between childhood trauma and depression is shown in Figure 2. With the changes in the level of resilience, the slope became flattened. At the highest level, the effect was insignificant, indicating that resilience attenuated the childhood trauma effect on depression.

The interacting effect of resilience with childhood trauma on the outcome of PTSD symptoms was analyzed using the same procedure as that used on depressive symptoms. However, the results revealed no significant interaction, regardless of whether covariates were included. The same covariates were included as those related to depressive symptoms. See Table 8. 

## 4. Discussion

This study stands among the first in Thailand to comprehensively examine the relationships between early-life adversity and current mental health outcomes in older adults within clinical settings. Recent studies on older adults have focused on attachment and loneliness [64,65], perceived social support [66,67], suicidality [23,68,69], and traumatic experiences such as elder abuse [16] relating to a particular mental health outcome, i.e., depression. PTSD among the older aged population, particularly for clinical populations, is grossly understudied. The current study initially provided the importance of PTSD among this population regarding traumatic life events across the lifespan. It confirms the presence of current PTSD symptoms in a significant percentage (11.4%) of older people in a clinical setting. The rate of probable depression (11.4%) was lower than figures reported before and during the COVID-19 pandemic in Thailand [23,64], while the prevalence of probable PTSD (11.4%) exceeded that found in long-term care settings [27].

Set against a backdrop of demographic transformation and the vulnerabilities associated with aging, this investigation primarily sought to determine whether resilience serves as a buffer against the effects of childhood trauma on depressive and PTSD symptoms in late life. In contributing to the broader discourse on the enduring impact of early adversity, the results show that both childhood and lifetime trauma are significantly and positively associated with depression and PTSD symptoms, consistent with global research on older adults [4,5,6,7,32,33,34,70]. The prevalence of childhood trauma (40.3%) and lifetime trauma (58.2%) in this cohort mirrors earlier findings, affirming that adverse experiences are shared and clinically salient [4,5,71].

Crucially, the analysis revealed that resilience moderates the relationship between childhood trauma and depressive symptoms, supporting the central hypothesis. Specifically, resilience served to attenuate the negative effect of childhood trauma on depression, explaining 36% of the variance in depressive symptoms after accounting for key covariates. This is in line with the growing body of evidence highlighting resilience as a potent protective factor for mental health, even among those carrying significant adversity histories. However, this buffering effect of resilience did not extend to PTSD symptoms in the present sample. It is plausible that in older adulthood, especially when PTSD symptoms are chronic or subthreshold, resilience developed throughout life is less effective in mitigating these symptoms. The findings support prior work suggesting that resilience in the aftermath of trauma does not equate to immunity from psychopathology; some residual PTSD symptoms may persist despite high resilience [45].

Resilience itself is increasingly understood as a dynamic, multidimensional construct influenced by factors such as personality, socioeconomic resources, and sociocultural context [44,45]. In this study, higher educational attainment was linked to greater resilience, consistent with the existing literature, and suggests that lifelong learning and cognitive resources may enhance psychological adaptation [48,49]. However, the nuanced relationship of resilience with different mental health outcomes was evident: while higher resilience buffered against depression, it showed variable associations with PTSD, suggesting that protective factors may operate differently across disorders. The resilience portfolio model, which emphasizes regulation, social connectedness, and meaning making, may help explain why resilience had a stronger protective role for depression as compared to PTSD in this setting [50].

Patterns of association between sociodemographic, clinical, and trauma-related variables further illuminate the complex landscape of mental health in later life. Higher education and income were associated with greater resilience and lower depression but, interestingly, also correlated positively with PTSD symptoms. In contrast, poor family relationships, exposure to recent or lifetime trauma, family psychiatric history, and recent falls were all linked to heightened depression and PTSD, reinforcing the need for holistic, trauma-informed care approaches in geriatric populations [48,49,72]. The strong correlation between depression and PTSD symptoms is aligned with the Hierarchical Taxonomy of Psychopathology (HiTOP) approach, which situates PTSD and depression as closely related within the internalizing spectrum [73], and is further substantiated by international data showing substantial comorbidity between these disorders in older adults [74,75]. Such findings may account for why resilience only appears to buffer depression and not the frequently overlapping symptoms of PTSD.

### 4.1. Implications and Future Research

This study highlights the importance of routine screening for depression and PTSD in older adults, especially those with a history of trauma. Utilizing validated screening tools and structured clinical interviews can facilitate early detection and prompt intervention. The findings underscore that childhood trauma remains a significant influence on mental health and resilience, even decades later. Although resilience helps mitigate depression, its limited effect on PTSD highlights the need for trauma-informed and individualized care strategies tailored to the varied backgrounds of older adults. Future research should further examine resilience, with an emphasis on cultural adaptation, emotion regulation, social connectedness, and trauma types related to PTSD development. Longitudinal designs are essential to understand better causal relationships and the evolving nature of resilience from midlife to old age.

### 4.2. Limitations

Several limitations should be noted. The cross-sectional design precludes causal inference and limits understanding of change over time; incorporating longitudinal follow-up would address this gap and clarify symptom trajectories. Reliance on retrospective self-report data introduces potential recall bias, and the lack of triangulation with clinician assessments, caregiver observations, or biomarkers reduces the robustness of the data. Important variables such as personality traits and coping styles were also not assessed. Despite these constraints, this study advances understanding of mental health among trauma-exposed older Thais and underscores the value of resilience and psychosocial factors in geriatric care. These insights provide a foundation for developing integrated, trauma-informed interventions and guiding future research on psychological adaptation in aging populations.

## 5. Conclusions

This study reveals that both childhood and lifetime trauma significantly predict depression and PTSD among older Thai adults, while resilience explicitly buffers the impact of childhood trauma on depression. The absence of a resilience effect on PTSD symptoms highlights the need for multifaceted, trauma-informed interventions in geriatric care. Our findings emphasize the value of integrating resilience building and culturally sensitive strategies into practice. Future research should adopt longitudinal and mixed methods approaches to further elucidate how resilience mechanisms operate in diverse older populations. By bridging trauma and aging research, this work advances understanding of psychological adaptation and informs both theory and clinical care.

## Figures and Tables

**Table 1:** Sociodemographic characteristics of participants (n = 201).

Characteristics	N (%) or Mean (SD)
Age	68.56 (±5.0)
60–69 years	118 (58.7%)
70–79 years	78 (38.8%)
80 and above	5 (2.5%)
Sex	
Female	141 (70.1%)
Male	60 (29.9%)
Religion	
Buddhist	194 (96.5%)
Non-Buddhist	7 (3.5%)
Education	
No education	3 (1.5%)
Primary education	50 (24.9%)
Secondary education	56 (27.9%)
Diploma	3 (1.5%)
Bachelor’s degree	64 (31.8%)
Master’s degree and above	25 (12.4%)
Marital status	
Single	12 (6.0%)
Married	140 (69.7%)
Divorced	19 (9.5%)
Widowed	30 (14.9%)
No. of household member	
1	9 (4.5%)
3 or less	99 (49.3%)
4 or more	93 (45.3%)
Living status	
Living alone	23 (11.4%)
Living with family	173 (86.1%)
Living with others	5 (2.5%)
Family relationship	
Good	159 (79.1%)
Average	42 (20.9%)
Poor	0
Monthly income	
5000 or less	59 (29.4%)
Between 5000 and 10,000	28 (13.9%)
10,000 and above	114 (56.7%)
Family history of psychiatric condition	
Yes	16 (8.0%)
No	185 (92.0%)
Medical coverage	
Gov. official	168 (83.6%)
30-baht scheme	20 (10.0%)
Social security scheme	5 (2.5%)
Health insurance	2 (1.0%)
Self-paid	6 (3.0%)
Alcohol consumption in the past month	
None	175 (87.1%)
Less than 3 times a week	20 (10.0%)
3–5 times a week	5 (2.5%)
More than 5 times a week	1 (0.5%)
Smoking/tobacco use in the past month	
No	196 (97.5%)
Yes	5 (2.5%)
Cannabis use in the past month	
No	196 (97.5%)
Yes	5 (2.5%)
History of fall in the past month	
Yes	6 (3.0%)
No	195 (97%)
History of physical comorbidities	
Yes	127 (63.2%)
No	74 (36.8%)
History of psychiatric comorbidities	
Yes	12 (6%)
No	189 (94%)
Childhood trauma	
No	120 (59.7%)
Yes	81 (40.3%)
Lifetime trauma	
No	84 (41.79%)
Yes	117 (58.21%)
Resilience Inventory-RI (9–45)	33.77 (±10.510)
Thai Geriatric Depression Scale 6 (TGDS-6)	0.52 (±0.94)
Depression prevalence	23 (11.4%) 95% CI: 7–17%
PTSD Check List for Civilian (PCL-C) (17–85)	27.70 (±10.62)
PTSD prevalence	23 (11.4%) 95% CI: 7–17%

Note: Physical comorbidity, having at least two diagnosed chronic diseases, i.e., hypertension, diabetes, hyperlipidemia/dyslipidemia, chronic obstructive pulmonary disease. Psychiatric comorbidity: having at least two diagnosed psychiatric conditions, i.e., depression, bipolar disorders, panic disorder, psychosis, generalized anxiety disorder (GAD), alcohol/substance use disorder, other. The cut-off point for depression is ≥2, and for PTSD is ≥42.

**Table 2:** Differential test of scores in resilience, depression, and PTSD symptoms by sociodemographic characteristics and traumatic experience (N = 201).

	Characteristics	Resilience Mean (SD)	Effect Size	Depression Score Mean (SD)	Effect Size	PTSD Symptoms Mean (SD)	Effect Size
Age	60–69 years	33.93 (10.13)	*η*^2^*_p_* = 0.005	0.55 (0.95)	*η*^2^*_p_* = 0.026	27.57 (9.61)	*η*^2^*_p_* = 0.050
	70–79 years	33.03 (10.87)		0.42 (0.83)		26.96 (10.78)	
	80 and above	37.20 (6.34)		1.4 (1.95)		42.40 (20.45)	
Sex	Female (141)	32.59 (10.48)	Cohen’s *d* = 0.36 *	0.59 (0.98)	Cohen’s *d* = 0.24	27.43 (11.11)	Cohen’s *d* = 0.089
	Male (60)	36.18 (9.60)		0.37 (0.82)		28.35 (9.43)	
Education	<up to secondary	32.86 (10.50)	*η*^2^*_p_* = 0.14 ***	0.55 (0.98)	Cohen’s *d* = 0.19	27.48 (10.78)	Cohen’s *d* = 0.14
	>Above secondary	38.06 (8.21)		0.39 (0.72)		28.90 (9.77)	
Monthly income	5000 or less	30.41 (11.68)	*η*^2^*_p_* = 0.073 **	0.66 (0.99)	*η*^2^*_p_* = 0.06 **	26.24 (11.29)	*η*^2^*_p_* = 0.120
	Between 5000 and 10,000	30.64 (11.13)		1.07 (1.36)		28.43 (12.59)	
	10,000 and above	36.09 (8.70)		0.32 (0.71)		28.28 (9.74)	
Marital status	Single	37.42 (5.43)	*η*^2^*_p_* = 0.021	0.58 (1.00)	*η*^2^*_p_* = 0.020	30.50 (10.82)	*η*^2^*_p_* = 0.034
	Married	33.99 (10.29)		0.43 (0.84)		26.56 (9.04)	
	Divorced	33.53 (11.56)		0.95 (1.31)		32.63 (15.15)	
	Widowed	30.70 (10.94)		0.67 (1.06)		28.77 (13.14)	
Family relationship	Good	33.94 (10.56)	Cohen’s *d* = 0.13	0.42 (0.83)	Cohen’s *d* = 0.46 *	26.10 (8.34)	Cohen’s *d* = 0.62 **
	Average	32.59 (9.47)		0.90 (1.23)		33.76 (15.31)	
Family history of psychiatric condition	Yes	36.81 (8.79)	Cohen’s *d* = 0.36	1.31 (1.62)	Cohen’s *d* = 0.67	41.00 (19.13)	Cohen’s *d* = 0.97 **
	No	33.39 (10.34)		0.45 (0.83)		26.55 (8.72)	
Alcohol consumption in the past month	None	33.30 (10.55)	*η^2^_p_* = 0.012	0.53 (0.94)	*η^2^_p_* = 0.017	27.55 (10.87)	*η^2^_p_* = 0.0060
	Less than 3 times a week	35.30 (8.96)		0.55 (1.10)		29.60 (9.44)	
	3–5 times a week	37.6 (7.27)		0.20 (0.45)		26.80 (6.53)	
	More than 5 times a week	44 (0.00)		1 (0.00)		20 (0.00)	
Smoking/Tobacco use in the past month	No	33.66 (10.38)	Cohen’s *d* = 0.01	0.48 (0.89)	Cohen’s *d* = 1.19 **	27.60 (10.64)	Cohen’s *d* = 0.38
	Yes	33.60 (9.50)		2.00 (1.58)		31.60 (10.26)	
History of fall in the past month	No	33.89 (10.14)	Cohen’s *d* = 0.61	0.50 (0.92)	Cohen’s *d* = 0.71 *	27.27 (9.67)	Cohen’s *d* = 0.76
	Yes	26.17 (14.59)		1.33 (1.37)		41.83 (25.28)	
History of physical comorbidities	No	33.32 (10.59)	Cohen’s *d* = 0.05	0.54 (0.86)	Cohen’s *d* = 0.03	27.46 (9.39)	Cohen’s *d* = 0.04
	Yes	33.86 (10.22)		0.51 (0.99)		27.84 (11.31)	
History of psychiatric comorbidities	No	33.84 (10.37)	Cohen’s *d* = 0.29	0.42 (0.82)	Cohen’s *d* = 1.64 ***	26.58 (8.86)	Cohen’s *d* = 1.28 ***
	Yes	30.92 (9.78)		2.17 (1.27)		45.33 (18.71)	
Childhood trauma	No trauma	33.73 (11.15)	Cohen’s *d* = 0.01	0.31 (0.66)	Cohen’s *d* = 4.61 *	24.78 (7.47)	Cohen’s *d* = 0.46
	At least one trauma	33.63 (9.90)		0.64 (1.05)		29.33 (11.74)	
Lifetime trauma	No trauma	32.24 (11.49)	Cohen’s *d* = 0.23	0.33 (0.79)	Cohen’s *d* = 0.36 *	23.99 (7.11)	Cohen’s *d* = 0.65 ***
	At least one trauma	34.68 (9.34)		0.66 (1.02)		30.37 (11.88)	

* *p* < 0.05, ** *p* < 0.01, *** *p* < 0.001, *η*^2^*_p_* = partial Eta.

**Table 3:** Spearman’s correlation between sociodemographic and measuring variables.

	1	2	3	4	5	6	7	8	9	10	11	12	13	14	15
1. Age in years	-														
2. Sex	0.05	-													
3. Religion	−0.05	0.12	-												
4. Education	−0.03	0.19 **	−0.03	-											
5. Marital status	0.16 *	−0.13	0.01	−0.16 *	-										
6. Living status	−0.13	−0.05	−0.12	−0.06	−0.12	-									
7. Family relationship	0.04	−0.18 *	−0.04	−0.20 **	0.16 *	−0.18 *	-								
8. Monthly income	0.02	0.32 **	0.06	0.61 **	−0.24 **	−0.01	−0.22 **	-							
9. Medical coverage	−0.07	0.06	0.09	−0.35 **	0.05	0.03	0.22 **	−0.27 **	-						
10. Alcohol used in the past month	−0.07	0.21 **	0.07	0.14 *	−0.08	−0.11	−0.09	0.22 **	0.04	-					
11. Childhood trauma	0.07	0.04	−0.09	−0.06	0.08	−0.18 *	0.14 *	−0.01	0.05	0.06	-				
12. Lifetime trauma	0.19 **	0	−0.06	0.04	0.08	0.13	0.06	0.06	−0.02	−0.02	0.04	-			
13. Resilience	−0.03	0.18 *	−0.01	0.37 **	−0.12	0.04	−0.09	0.24 **	−0.11	0.09	−0.08	0.13	-		
14. Depression	−0.02	−0.13	−0.02	−0.15 *	0.11	−0.06	0.19 **	−0.21 **	0.08	−0.01	0.25 **	0.21 **	−0.25 **	-	
15. PTSD symptoms	0.03	0.08	0.05	0.15 *	0.05	−0.04	0.22 **	0.14 *	0.11	0.07	0.14 *	0.38 **	0.08	0.35 **	-

Note: N = 201, * *p* < 0.05, ** *p* < 0.01. Sociodemographic variables were categorized and measuring variables were continuous.

**Table 4:** Point biserial correlation between clinical condition and measuring variables (N = 201).

	1	2	3	4	5	6	7	8	9	10	11
1. Family HO psychiatric condition	-										
2. Smoking/tobacco use in the past month	0.05	-									
3. Cannabis use in the past month	0.05	0.18 *	-								
4. History of fall in the past month	0.16 *	−0.03	−0.03	-							
5. Physical comorbidity	0.15 *	−0.08	0.06	0.07	-						
6. Psychiatric comorbidity	0.31 **	−0.04	−0.04	0.08	0.10	-					
7. Childhood trauma	0.1	−0.07	−0.07	0.08	−0.08	0.27 **	-				
8. Lifetime trauma	0.26 **	0.04	−0.03	0.20 **	0.06	0.13	0.11	-			
9. Resilience	0.09	−0.00	0.10	−0.13	0.03	−0.07	−0.15 *	0.16 *	-		
10. Depression	0.25 **	0.25 **	0.22 **	0.15 *	−0.01	0.44 **	0.43 **	0.23 **	−0.22 **	-	
11. PTSD symptoms	0.37 **	0.06	0.05	0.23 **	0.02	0.42 **	0.31 **	0.40 **	0.15 *	0.51 **	-

Note: N = 201. * *p* < 0.05, ** *p* < 0.01. Clinical condition: family history of psychiatric condition, history of fall, and physical and psychiatric comorbidity were dichotomized (Yes/No). Measuring variables are continuous. Physical comorbidity: having at least two diagnosed chronic diseases (hypertension, diabetes, hyperlipidemia/dyslipidemia, COPD, and other). Psychiatric comorbidity: having at least two diagnosed psychiatric conditions (depression, bipolar disorders, panic disorder, psychosis, GAD, AUD/SUD, and other).

**Table 5:** Mean, standard deviation, and correlation coefficients between study variables.

Measuring Variables	Mean (SD)	1	2	3	4	5
1. Childhood trauma (0–25)	0.76 (1.27)	-				
2. Lifetime trauma (0–16)	1.31 (1.78)	0.11	-			
3. Resilience (9–45)	33.66 (10.34)	−0.15 *	0.16 *	-		
4. Depression (0–6)	0.53 (0.943)	0.43 **	0.23 **	−0.22 **	-	
5. PTSD symptoms (17–85)	27.70(10.62)	0.31 **	0.40 **	0.15 *	0.51 **	-

SD = standard deviation, ** *p* < 0.01, * *p* < 0.05.

**Table 6:** The moderating effect of resilience between childhood trauma and depression.

Model		Coeff.	SE	t	*p*-Value	LLCI	ULCI
1	Constant	0.37	0.05	7.63	<0.001	0.273	0.463
R^2^ = 0.12	Childhood trauma (X)	0.17	0.04	4.29	<0.001	0.102	0.254
MSE = 0.475	Resilience (W)	−0.01	0.01	−2.08	0.039	−0.019	0.000
2	Constant	0.35	0.05	7.36	<0.001	0.260	0.450
R^2^ = 0.14	Childhood trauma (X)	0.14	0.04	3.62	<0.001	0.065	0.221
MSE = 0.460	Resilience (W)	−0.01	0.00	−1.76	0.081	−0.018	0.001
	Interaction (X × W)	−0.01	0.00	−2.17	0.032	−0.013	−0.001
3	Constant	0.38	0.63	0.603	0.547	−0.862	1.621
R^2^ = 0.36	Childhood trauma (X)	0.08	0.04	2.14	0.034	0.007	0.160
MSE = 0.364	Resilience (W)	−0.01	0.00	−1.51	0.133	−0.016	0.002
	Interaction (X × W)	−0.01	0.00	−3.00	0.003	−0.015	−0.003

Note: Coeff = unstandardized coefficient; SE = standard error; LLCI = lower level of confidence interval; ULCI = upper level of confidence interval. Controlled variables are age, sex, education, income, family relationship, alcohol, smoking, history of physical and psychiatric disorders, and lifetime trauma.

**Table 7:** Hierarchical regression models testing the moderating effect of resilience on the relationship between childhood trauma and depression.

Model	Predictors	R^2^	∆R^2^	f^2^	F	*p*-Value
1	X, M	0.12	-	-	12.99	0.000
2	X, M, X × M (interaction)	0.14	0.02	0.023	10.38	0.000
3	X, M, X × M (with covariates)	0.36	0.24	0.375	6.50	0.000

Note: R^2^ = coefficient of determination, f^2^ = Cohen’s effect size, F-test for overall model fit.

**Table 8:** Moderating effect of resilience between childhood trauma and PTSD symptoms, controlling for age, sex, education, income, family relationship, alcohol, smoking, history of physical and psychiatric disorders, and lifetime trauma.

Model		Coeff.	SE	t	*p*-Value	LLCI	ULCI
1	Constant	27.70	0.70	39.52	<0.001	26.319	29.084
R^2^ = 0.09	Childhood trauma (X)	2.83	0.56	5.04	<0.001	1.723	3.936
MSE = 102.7	Resilience (W)	0.21	0.07	2.99	0.003	0.070	3.341
2	Constant	27.84	0.70	39.50	<0.001	26.446	29.225
R^2^ = 0.14	Childhood trauma (X)	3.05	0.58	5.27	<0.001	1.908	4.193
MSE = 98.16	Resilience (W)	0.19	0.07	2.75	0.007	0.054	0.327
	Interaction (X × W)	0.07	0.05	1.48	0.142	−0.023	0.158
3	Constant	16.01	8.56	1.871	0.063	−0.876	32.895
R^2^ = 0.45	Childhood trauma (X)	1.65	0.53	3.11	0.002	0.601	2.692
MSE = 67.32	Resilience (W)	0.15	0.06	2.41	0.017	0.027	0.270
	Interaction (X × W)	0.02	0.04	0.47	0.634	−0.060	0.099

Note: Coeff = unstandardized coefficient; SE = standard error; LLCI = lower level of confidence interval; ULCI = upper level of confidence interval.

## Data Availability

The datasets generated and/or utilized for the analysis in the current study are not publicly available for the reason of ethics approval but are available from the corresponding author on reasonable request.

## Abbreviations

The following abbreviations are used in this manuscript:

ASEAN	Association of Southeast Asian Nations
PTSD	Post-Traumatic Stress Disorder
DSM	Diagnostic and Statistical Manual of Mental Disorders
MBI	Mindfulness-Based Intervention
MDD	Major Depressive Disorder
